# DFT and TD-DFT Study of the Chemical Effect in the SERS Spectra of Piperidine Adsorbed on Silver Colloidal Nanoparticles

**DOI:** 10.3390/nano12172907

**Published:** 2022-08-24

**Authors:** Francesco Muniz-Miranda, Alfonso Pedone, Maria Cristina Menziani, Maurizio Muniz-Miranda

**Affiliations:** 1 Department and Geological Sciences (DSCG), University of Modena and Reggio Emilia, Via Campi 103, 41125 Modena, Italy; 2 Department of Chemistry “Ugo Schiff” (DiCUS), University of Florence, Via Lastruccia 3, 50019 Sesto Fiorentino, Italy

**Keywords:** DFT, piperidine, Ag nanoparticles, SERS, chemical enhancement

## Abstract

The surface-enhanced Raman scattering (SERS) spectra of piperidine adsorbed on silver/chloride colloids were studied by a combined density functional theory (DFT)/time dependent DFT (TD-DFT) approach. The mechanism of chemical enhancement on the Raman signals is due to at least two contributions: the first comes from the changes in the molecular force constants and the dynamic polarizabilities of the normal modes, when the molecule is chemisorbed. DFT calculations satisfactorily reproduce the SERS spectra of piperidine adsorbed on silver, showing that the species formed on the silver particle is a complex formed by a deprotonated piperidine linked to a silver cation. A second contribution to the SERS chemical enhancement is due to a resonance Raman effect occurring when the wavelength of the Raman excitation falls within the electronic excitation band of the molecule/metal complex. Actually, the SERS spectra of piperidine show a significant dependence on the wavelength of the laser excitation, with a marked enhancement in the green-light region. TD-DFT calculations on the most-probable complex explain this behavior, because a strong excitation band of the complex is calculated in the green spectral region. This pinpoints that a resonance between the exciting radiation and the absorption band of this complex is responsible for this enhancement effect.

## 1. Introduction

SERS (surface-enhanced Raman scattering) effect [1,2] provides huge intensification of the Raman signal of molecules adsorbed on nanostructured surfaces of metals such as silver, gold, or copper. It can be attributed to the combined action of two mechanisms, one of electromagnetic type, due to the plasmonic excitation of metal, another of chemical type depending on the interactions between adsorbed molecules and active sites of the metal surface. The first mechanism requires two conditions: (i) the nanostructured substrate must be formed with a high optical reflectivity metal, that is, the imaginary part of the dielectric constant has to be very small in the spectral region of Raman excitation; (ii) the wavelength of the excitation radiation must occur within the plasmon band of metal nanoparticles, that is, it is necessary that the incident and scattering radiations are resonant with the plasmon excitation. Hence, the irradiated molecules adhering to metal undergo an electric field by several orders of magnitude larger than that at long distances from the surface, exhibiting enhancement factors of their Raman signals up to 10^7^. In this way, one can observe the Raman spectra of molecular sub-monolayers adhering to metal surfaces of appropriate roughness, also without the formation of chemical bonds between the molecules and metal. The electromagnetic contribution plays a predominant role for the SERS enhancement because the chemical mechanism usually improves the enhancement factor only up to 10^2^.

However, the chemical contribution plays an important role in the SERS spectral pattern, because strong ligand/metal interactions can provoke significant frequency shifts of the SERS bands with respect to the normal Raman spectra of the non-adsorbed molecules, as well as significant changes in the relative intensities. To observe a chemical SERS enhancement, it is necessary that the molecules approach up to distances of about 2 Angstroms to the metal surface, where defects are present as active sites in the chemisorption process. The chemical enhancement is due mostly to two contributions: one non-resonant (static), due to the perturbation in the electron density of the chemisorbed molecule, which can intensify the dynamic polarizabilities of some normal modes of the complex formed by ligand molecules and active sites of the metal surface; another of charge-transfer that can be evidenced as Raman resonance by varying the electrode potential or the exciting radiation. In particular, this resonance Raman effect occurs when the wavelength of the Raman excitation falls within the electronic excitation band of the molecule/metal complex. The first contribution, which affects both positions and relative intensities of the SERS bands differently, can be simulated well by DFT (density functional theory) calculations on the basis of the ground state of the model systems as molecules linked to metal adatoms, also recently performed [3,4,5,6,7,8,9]. The second contribution could be analyzed instead within a time-dependent DFT approach, by calculating the excitation bands of the ligand/metal complex, which experimentally cannot be observed in the UV–visible absorption spectrum owing to the extremely low concentrations of the complex.

Here, we have studied the chemical effect in the SERS spectra of piperidine adsorbed on Ag colloidal nanoparticles. Piperidine is a saturated heterocyclic molecule, which can chemically interact with metal through the lone pair of the nitrogen atom. However, this investigation is made more complex by the fact that piperidine can occur in liquid state in two different conformations, when the hydrogen atom linked to nitrogen is either in equatorial or in axial conformation with respect to the average plane of the molecule. In the gas phase, the equatorial conformation was found more stable [10], as well as in non-polar solvents. In Figure S1 of the Supplementary Materials the optimized structure of piperidine in equatorial conformation is shown. In polar solvents, instead, the axial conformer could be more stable [11]. This suggests the possibility that the molecule binds silver in aqueous colloidal suspension both in the axial position and in the equatorial position, due to rapid interconversion between conformers, as experimentally observed in dynamic NMR studies of piperidine in solution [12]. In addition, piperidine could adsorb on Ag colloidal nanoparticles in the deprotonated form by the effect of a surface reaction with the metal active sites. A DFT study, based on complex models of the molecule in neutral or anionic form, with equatorial or axial conformation, linked to one silver adatom, could effectively simulate the SERS spectrum of piperidine as regards the positions of the bands, their relative intensities and the frequency shifts observed with respect to the normal Raman spectrum. Moreover, if one of these complexes is found able to reproduce satisfactorily the SERS spectrum, the excitation bands of this complex can be obtained by the TD-DFT approach, in order to provide information on the resonance Raman effect, which could act in the chemical enhancement.

## 2. Materials and Methods

### 2.1. Sample Preparation

Silver hydrosols were prepared following the Creighton’s procedure [13], by adding AgNO_3_ (purity: 99.9999%, Sigma-Aldrich, St. Louis, MI, USA) to excess NaBH_4_ (purity: 99.9%, Aldrich), aged a week to prevent the formation of reduction products [14]. NaCl (purity: ≥99%, Aldrich), was added to the Ag colloid in 10^−3^ M concentration, in order to improve SERS enhancement. Piperidine (purity: ≥99%, Alfa Aesar, Ward Hill, MA, USA) was added to the colloidal sample with 10^−5^ M final concentration.

### 2.2. Raman Spectroscopy

Raman and SERS spectra were recorded using the 514.5 nm line of an argon ion laser, and a Jobin–Yvon HG2S monochromator equipped with a cooled RCA-C31034A photomultiplier (Horiba-Jobin-Yvon, Tokyo, Japan). For the SERS spectra also the 457.9 nm and 488.0 nm lines of the Ar^+^ laser and the 568.2 nm and 647.1 nm lines of a Kr^+^ laser were used as exciting radiations. All spectra were corrected to account for monochromator and photomultiplier efficiency and normalized to the Raman scattering of ethanol as external standard.

### 2.3. UV–Vis Extinction Spectroscopy

UV−vis absorption spectra of colloidal suspensions were obtained in the 200–800 nm region by using a Cary 5 Varian spectrophotometer.

### 2.4. Density Functional Theory Calculations

DFT calculations were carried out using the GAUSSIAN 09 package [15]. Optimized geometries were obtained at the DFT level of theory, employing the widely adopted Becke 3-parameters hybrid exchange functional (B3) combined with the Lee–Yang–Parr correlation functional (LYP) [16,17], along with the Lanl2DZ basis set and pseudopotential [18,19,20]. The aqueous solvent was modeled within the polarizable continuum model, adopting the integral equation formalism model [21]. The effects of dispersion forces were taken into account employing Grimme’s D3 correction along Becke–Johnson damping [22]. All parameters were allowed to relax and all calculations converged toward optimized geometries corresponding to energy minima, as revealed by the lack of negative values in the frequency calculation. No empirical scaling factor was used. For piperidine (both neutral and in anionic form), the spin multiplicity was put equal to 1, as in the case of the Ag^+^ cation, whereas to model the Ag(0) atom, we resorted to unrestricted calculations with spin multiplicity equal to 2. In TD-DFT calculations we considered up to 12 excited states of both singlet and triplet multiplicity (these latter, as expected, have vanishing oscillator strength).

For the sake of completeness, we also checked two other levels of theory to investigate the most likely complex: (1) adopting a mixed basis set composed by Pople’s 6-311++G(d, p) for the organic molecule and Lanl2DZ for the silver cation; (2) adopting the CAM-B3LYP [23]/Lanl2DZ level of theory to better describe long-range electronic correlation.

The binding energies for the piperidine-silver bonds of the different models of the complex were calculated taking into account the BSSE (basis-set superposition error) correction: in these cases, however, no implicit solvent by polarizable continuum model was used.

## 3. Results and Discussion

### 3.1. Raman/SERS Spectra

**Figure 1:**
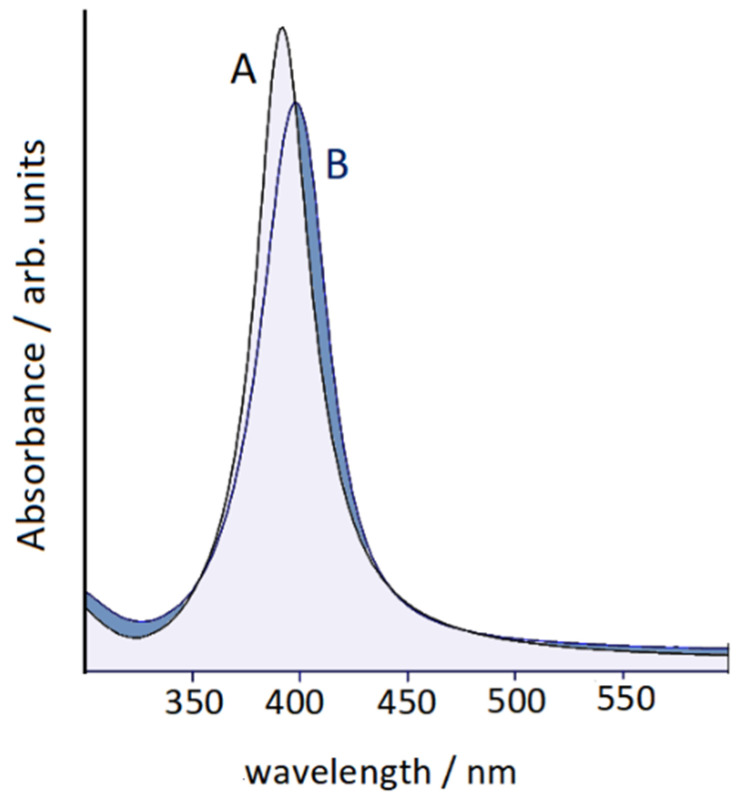
UV–vis absorption spectra of Ag colloids without (**A**) and with (**B**) the presence of piperidine.

Piperidine was added in 10^−5^ M concentration to a colloidal suspension of silver nanoparticles, activated by the presence of chloride anions, which provide a strong increase in the SERS signal of the adsorbed organic molecules. In this regard, it is important to underline that the chemical enhancement mechanism plays an essential role in the SERS spectra when the surface of the silver nanoparticles is activated by the presence of halide anions, in particular chlorides. These latter, when adsorbing on the metal, promote the formation of efficient active sites [24,25,26,27], able to chemically bind molecules that exhibit localized electronic charges, mainly due to the presence of heteroatoms. Consequently, a marked increase in the SERS response occurs. The UV–vis absorption spectra of Ag colloids with and without the presence of piperidine are reported in Figure 1. The silver colloid in the presence of chloride showed a plasmonic band around 390 nm, without evidence of particle aggregation, because of the repulsion of the negative charges due to the adsorption of chloride anions on the surface of the silver nanoparticles. Even by adding piperidine, apart from a small shift of the plasmonic band, no aggregation was observed, in the absence of secondary plasmonic bands at longer wavelengths.

**Figure 2:**
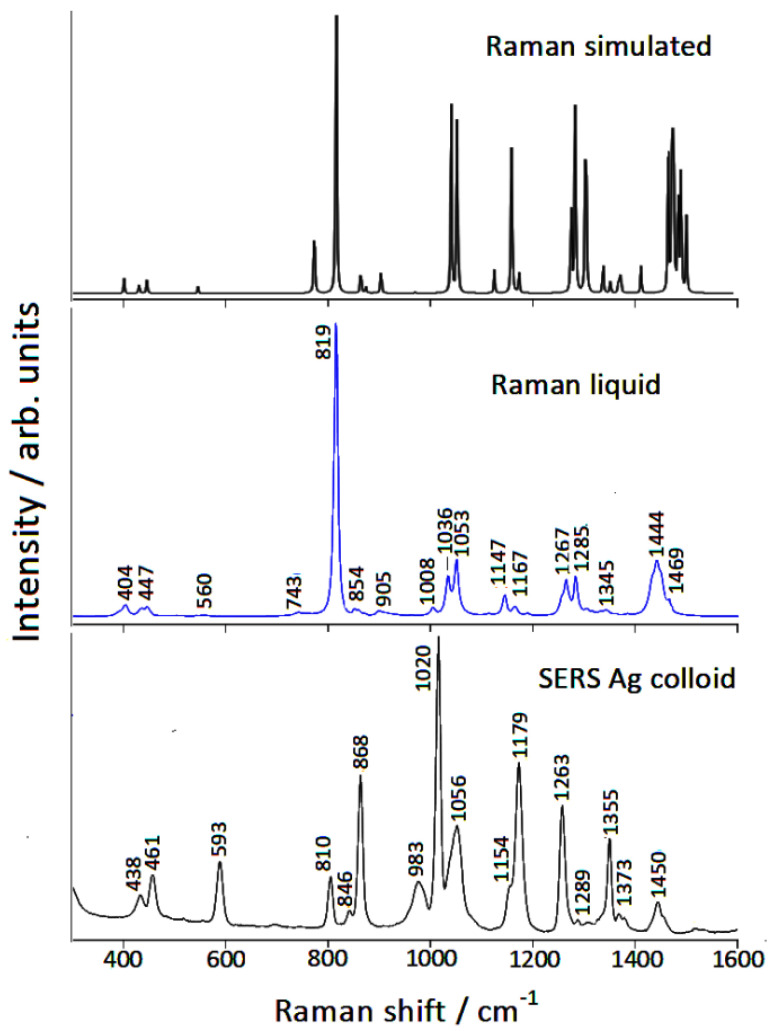
Raman spectra of piperidine.

The SERS spectrum of piperidine adsorbed on Ag nanoparticles is reported in Figure 2, compared with the normal Raman spectrum of the molecule in the liquid phase. The observed SERS spectrum was quite different from the Raman one, in both band positions and relative intensities. These differences can be analyzed by DFT calculations. First, we tested the reliability of our calculation method on the isolated molecule, whose simulated Raman spectrum reproduced the normal Raman spectrum well in both frequencies and intensities, as evidenced in Table 1 and in Figure 2. It should be emphasized that this good agreement was obtained by considering the molecule in its equatorial conformation, as expected in the absence of strong interactions with the surrounding environment. In addition, the calculated structural parameters were similar to the experimental ones [28], as shown in Figure S1 of the Supplementary Materials.

### 3.2. DFT Calculations

A DFT study could effectively reproduce the SERS spectrum of piperidine by considering model complexes where the molecule is linked to silver adatoms or adclusters through the lone pair of the nitrogen atom. In the past, it was shown that in the colloids activated by halide anions the surface-active sites were positively charged, so the SERS spectral patterns often appeared very similar to those shown by normal Raman spectra of the corresponding Ag (I) coordination compounds [30,31,32,33,34,35,36]. Therefore, in these cases, DFT calculations based on a simplified adsorption model with the molecule linked to a single silver adatom could enable the satisfactory reproduction of both positions and relative intensities of the SERS bands. 

For this purpose, we have considered different complexes:

1. Molecule linked through the lone pair of nitrogen to a silver atom;
2. Molecule linked to a silver ion, because there can be positively charged active sites on the surface of Ag nanoparticles activated by coadsorbed chloride anions;
3. Piperidine deprotonated due to a surface reaction with a positive active site, even if the pKa of the molecule in water is 10.45 (at 25°) and the pH of the colloid is around 9; this possible reaction can be schematized as in Figure S2 of the Supplementary Materials.

**Figure 3:**
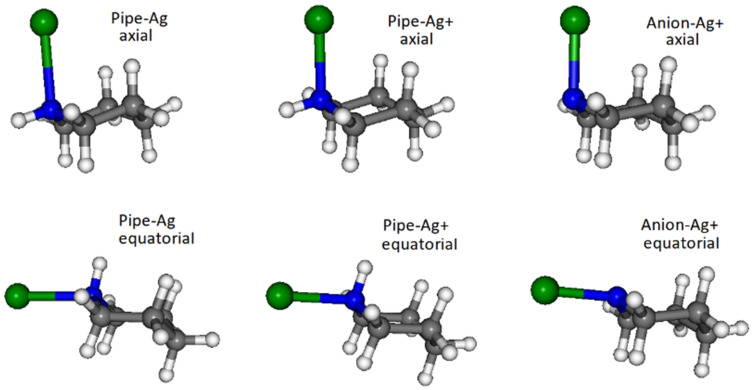
Piperidine/silver model complexes. Colors are standard CPK default, apart from the silver atom/ion which is depicted in green for better visualization.

For each of the three complexes, called Pipe-Ag, Pipe-Ag^+^ and Anion-Ag^+^, we considered the possibility that piperidine binds to silver both in the axial position and in the equatorial position, due to an interconversion between conformers. Thus, we performed DFT calculations to obtain structures and Raman spectra of silver complexes, as shown in Figure 3. The binding energies of the anion-Ag^+^ bonds were much larger (i.e., more negatively valued) than those corresponding to the complexes with the neutral molecule, whereas the energy differences between axial and equatorial conformations were almost negligible as reported.

**Figure 4:**
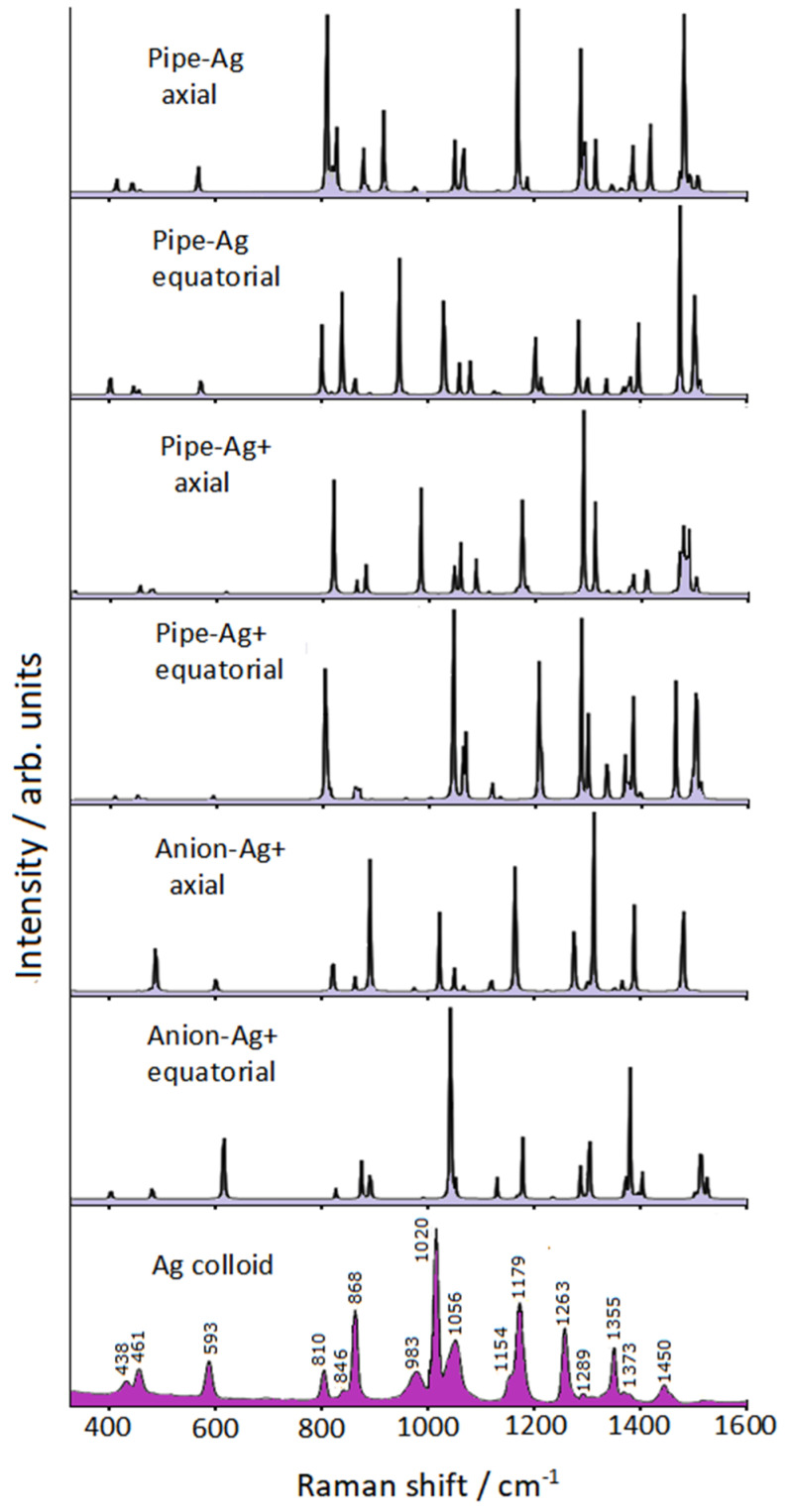
Raman spectra of silver/piperidine complexes compared with the SERS spectrum of piperidine in Ag colloid.

The Raman spectra calculated for these six possible complexes are reported in Figure 4 and compared with the SERS spectrum of piperidine adsorbed on silver colloid.

The complexes in axial conformation were not able to reproduce the SERS spectrum; in particular, the only complex capable of effectively simulating the observed SERS spectrum, as regards both band positions and intensities, was that in which the anion of deprotonated piperidine in equatorial conformation was linked to an Ag^+^ ion (Anion-Ag^+^ equatorial). Moreover, it should be noted that the strongest interaction between molecule and metal occurred for this complex, with the shortest N-Ag distance (2.111 Å) and the largest molecule→metal charge-transfer (−0.6932 |*e*|). In addition, the agreement between calculated Raman observed SERS frequencies was satisfactory, as shown in Table 1. Figure S3 of the Supplementary Materials shows the structure of the Anion-Ag^+^ equatorial complex, along with the normal modes corresponding to the prominent SERS bands, which were attributable to ring deformation modes. The N-Ag distance remained unchanged in the vibrations, except for the two bands at lower frequencies.

Furthermore, we performed further calculations to assess the robustness of our computational approach.

(1) We considered the Anion-Ag^+^ equatorial complex, identified as the most likely anchoring geometry, and we performed B3LYP calculations but with a mixed basis set made up of Pople’s 6-311++G(d, p) for the organic anion and LanL2DZ for the metal cation: we did this in order to check if a larger basis set could improve the results presented above.
(2) On the same complex, we also adopted a CAM-B3LYP/LanL2DZ level of theory to check if long-range electronic correlation (better captured by this functional) could improve the agreement with the experiments.
  In both cases, the latter agreement was actually worse than with the standard B3LYP/LanL2DZ approach used in most of this paper. Indeed, the average difference between computed and experimentally observed frequencies increased, and some intense SERS bands could not be assigned, as shown in Table S2 of the Supplementary Materials.
(3) We performed B3LYP/LanL2DZ calculations on piperidine anion linked to a larger adcluster (Ag_5_^+^) in order to better model the silver surface. This model did not show significant improvement in the agreement with SERS spectra, in comparison with calculations with a single Ag^+^. This result, reported in Table S3 of the Supplementary Materials, is not surprising because the local interaction between molecule and metal active-site is determinant for the SERS profile, as ascertained in different SERS studies of molecules chemisorbed on colloidal silver [7,9,25,37]. In fact, this corroborates the use of simple models to capture most of the chemical interaction relevant for SERS interpretation.

Finally, it must be emphasized that our SERS spectrum in the silver colloid activated with chloride anions was very similar to that recorded on Ag/AgCl electrode with KCl as electrolyte at potential V = 0 [38]. This is not surprising, because even on the electrode the chloride anions performed their SERS activation and the electrode at potential V = 0 was positively charged, as it was greater than the zero-charge potential [39].

### 3.3. TD-DFT Calculations

**Figure 5:**
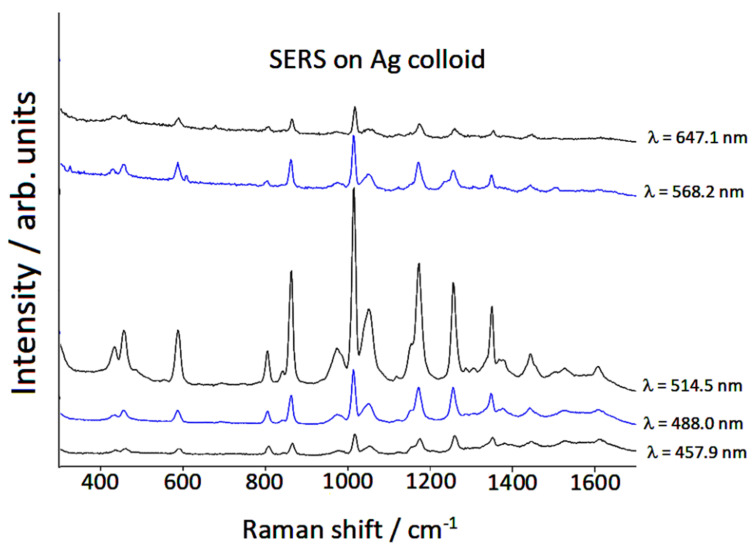
SERS spectra of piperidine by excitation with different laser lines.

We analyzed the resonant contribution of the chemical effect on the SERS spectra of piperidine by recording these latter employing different exciting laser lines. These spectra, reported in Figure 5, were normalized with respect to a common external reference and with respect to the instrumental efficiency that varied according to the exciting line used.

A marked intensification of the SERS signal by excitation in the region of green radiation (500–550 nm) was observed. This intensification cannot be attributed to the electromagnetic effect linked to plasmon resonance, because no plasmon band was observed in the green light region of the absorption spectrum of the Ag colloid in the presence of piperidine, as shown in Figure 1. Therefore, we can attribute this effect to the chemical enhancement mechanism, as a resonance Raman enhancement between the exciting radiation and the excitation band of the surface complex formed by chemical interaction of piperidine molecules with the active sites of the silver nanoparticles. Actually, the occurrence of this excitation band was not detected in the absorption spectrum, but this was reasonable, considering that in the Ag colloid there was a very low concentration of this complex, as a very minority amount with respect to the analytical concentration of added piperidine (10^−5^ M). On the other hand, it was evident that the chemical effect played a relevant role in the case of piperidine adsorbed on silver, since the SERS spectrum was very different from the normal Raman spectrum, in both positions and intensities of the bands.

**Figure 6:**
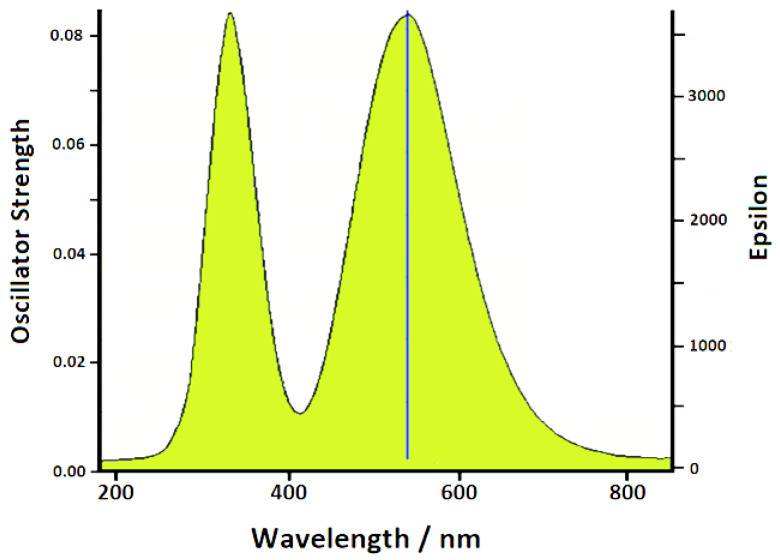
Calculated excitation spectrum of Anion-Ag^+^ equatorial complex.

Therefore, we performed a TD-DFT calculation on the Anion-Ag^+^ equatorial complex, which we selected as the most probable. A strong electronic excitation band occurred in the green region, as shown in Figure 6. As a conclusion, the marked enhancement observed in the SERS spectra by excitation in the green light region can be confidently attributed to a Raman resonance effect when the exciting radiation falls in the excitation band of the complex that forms on the colloidal surface. On the other hand, the other adsorption models did not show electronic transitions in the region of green radiation.

**Figure 7:**
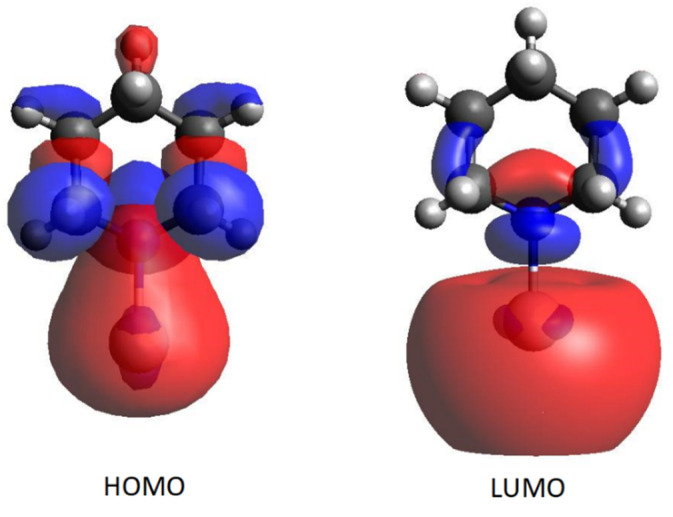
Calculated HOMO and LUMO orbitals of the Anion-Ag^+^ equatorial complex.

Finally, the transition calculated around 540 nm was totally attributed to the excitation between the HOMO and LUMO orbitals, which, as shown in Figure 7, strongly perturbs the electronic properties of the metal, as well as those of the molecular atoms.

## 4. Conclusions

We studied the SERS spectra of piperidine adsorbed on silver/chloride colloids by means of combined DFT and TD-DFT calculations, analyzing the chemical effect on the Raman enhancement of the molecule. This effect, in fact, is particularly important for silver colloids activated by the coadsorption of chloride anions, as it provides a marked interaction between the molecules and active sites of the metal surface. In the specific case of piperidine, the importance of this effect is shown by the marked differences observed between the normal Raman spectrum and the SERS spectrum of the molecule.

The chemical enhancement mechanism is due to at least two contributions, both related to the formation of molecule/metal complexes. The first contribution derives from the changes in both the molecular force constants and the dynamic polarizabilities of the normal modes, when the molecule is chemisorbed. Hence, significant differences occur in both positions and intensities of the observed SERS bands. In the present work, we analyzed these spectral features by DFT calculations, by considering model complexes with the piperidine molecules in two possible conformational situations, axial and equatorial, linked to neutral or positively charged silver adatom. We also took into account the possible deprotonation of piperidine when adsorbing on a positive metal surface. On the other hand, XPS studies previously ascertained that a significant amount of Ag (I) is really present on the surface of the silver nanoparticles activated by chloride ions [40]. Here, our DFT calculations allowed the simulation of satisfactory Raman and SERS spectra of piperidine, indicating the complex formed by a deprotonated molecule in equatorial conformation linked to a silver ion as the most probable species formed on the silver particle surface, which is characterized by a strong electronic charge-transfer from piperidine to metal. A second contribution to the SERS chemical enhancement is due to a resonance Raman effect that occurs when the wavelength of the Raman excitation falls within the electronic excitation band of the molecule/metal complex. Actually, the SERS spectra of piperidine show a significant dependence on the wavelength of the laser lines employed in the Raman excitation, with a marked enhancement in the region of the green radiation. In order to explain this spectral behavior, we carried out TD-DFT calculations on the complex identified as the most probable, which showed a strong excitation band in the green spectral region, totally attributable to a HOMO-LUMO transition. This indicates that a resonance between the exciting radiation and the absorption band of the complex is responsible for this enhancement effect.

As a conclusion, we showed how DFT calculations, along with a time-dependent approach, were able to account for the SERS spectra of piperidine, in particular for both the contributions to the chemical enhancement mechanism. In the future, a further improvement with experimental data may be achieved by adopting computationally more burdensome ab initio molecular dynamics simulations, along with specifically purposed tools, such as, for instance, the TRAVIS software [41] or approaches based on wavelet transformations [42].

## Figures and Tables

**Table 1:** Vibrational frequencies (cm^-1^) of both experimental and calculated spectra.

Infrared Liquid [29]	Raman Liquid	Calculated	SERS Ag Colloid	Calculated #
404	404	406		
432		436	438	452
445	447	452	461	478
546	560	550	593	611
743	743	779		
822	819	822	810	821
			846	868
859	854	871	868	884
906	905	909		
964		976		
1006	1008	1048	983	981
1035	1036	1059	1020	1032
1052	1053	1062	1056	1041
1115		1132		
1146	1147	1165	1154	1157
1164	1167	1166	1179	1166
1191		1180		
1258		1280		
1266	1267	1282	1263	1275
1285	1285	1290	1289	1290
1318		1310		
1332	1345	1345	1355	1367
1365		1360		
1386		1380	1373	1389
1436		1420		
1444	1444	1471		
1452		1479	1450	1494
1460		1482		
1468	1469	1491	1470	1498
1476		1497		

# Anion-Ag^+^ equatorial model.

## Data Availability

Data is contained within the article or supplementary material.

## Supplementary Materials

The following supporting information can be downloaded at: https://www.mdpi.com/article/10.3390/nano12172907/s1, Figure S1: DFT-optimized structure of piperidine, along with the experimental structural parameters; Figure S2: Possible reaction of piperidine with the positive active sites of the Ag surface; Figure S3: Calculated structure and normal modes of the Anion-Ag^+^ equatorial complex, corresponding to the most prominent experimental SERS bands; Table S1: Binding energies of the model shown in Figure 3 of the main manuscript; Table S2. Comparison of calculated Raman frequencies of the Anion-Ag^+^ equatorial model with different levels of theory and experimental SERS data; Table S3. Comparison of experimental and calculated frequencies for piperidine Anion linked to Ag^+^ and Ag_5_^+^. The four silver atoms on the plane were kept fixed during the structural relaxation.
