# Supplementary material for: DFT and TD-DFT Study of the Chemical Effect in the SERS Spectra of Piperidine Adsorbed on Silver Colloidal Nanoparticles
Source: Nanomaterials (Basel). 2022 Aug 24;12(17):2907. doi: 10.3390/nano12172907 (PMC9458020; doi:10.3390/nano12172907)

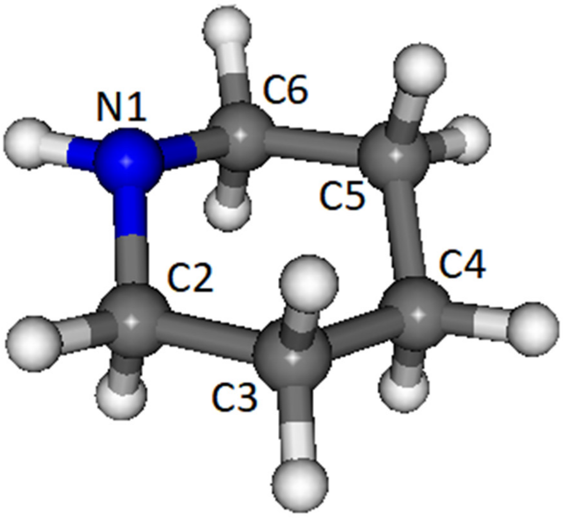

Distance (Å)	exp.	calc.
N1-C2	1.4613	1.469
N1-C6	1.4602	1.469
C2-C3	1.5207	1.531
C5-C6	1.5181	1.531
C3-C4	1.5189	1.536
C4-C5	1.5230	1.536

Angle (degree)	exp.	calc.
C2-N1-C6	111.04	111.8
N1-C2-C3	109.84	109.9
N1-C6-C5	109.96	109.9
C2-C3-C4	110.70	110.7
C4-C5-C6	110.21	110.7
C3-C4-C5	110.34	110.7

Figure S1. DFT-optimized structure of piperidine. Experimental structural parameters. Reprinted from Ref. [29].

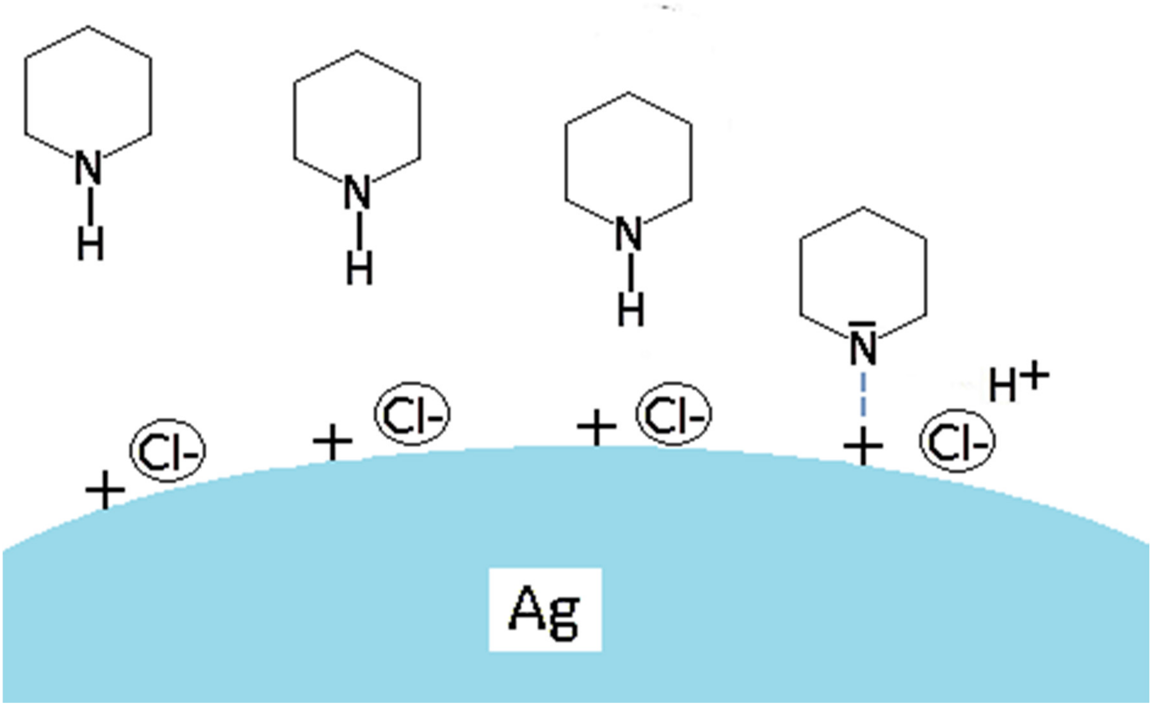

Figure S2. Possible reaction of piperidine with the positive active sites of the Ag surface.

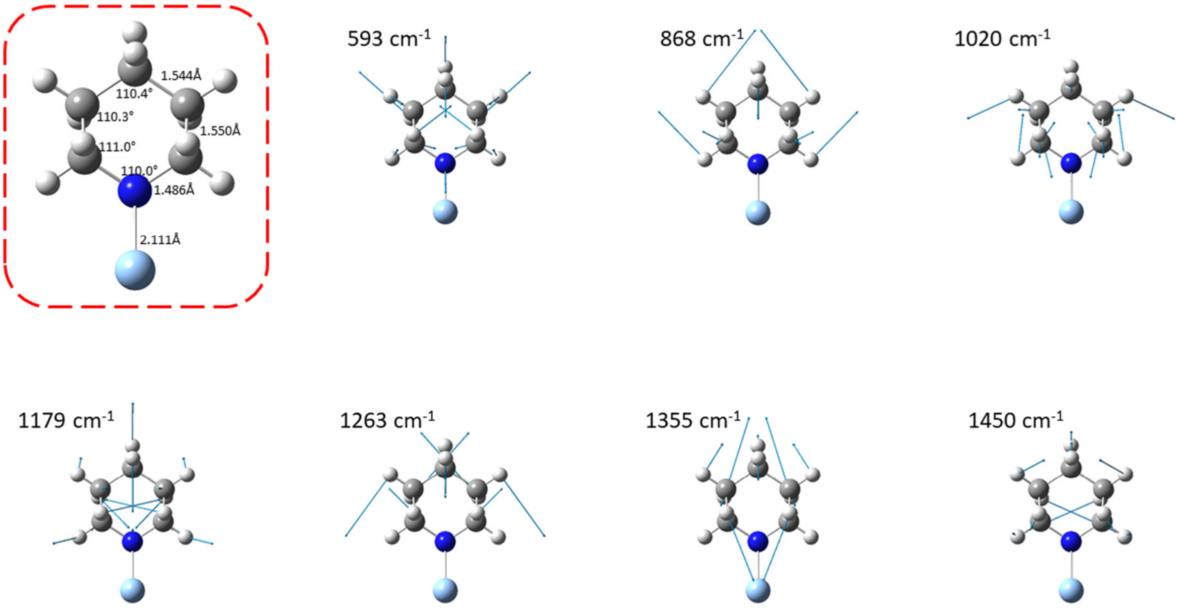

Figure S3. Calculated structure and normal modes of the Anion-Ag+ equatorial complex corresponding to the most prominent experimental SERS bands (see **Figure 2**).

Table S1. Binding energies of the model shown in **Figure 3** of the main manuscript.

**Binding energy**	**type of model**
-202.25 kcal/mole	Anion-Ag+ equatorial
-204.42 kcal/mole	Anion-Ag+ axial
-63.45 kcal/mole	Pipe-Ag+ equatorial
-65.19 kcal/mole	Pipe-Ag+ axial
-11.93 kcal/mole	Pipe-Ag equatorial
-11.09 kcal/mole	Pipe-Ag axial

Table S2. Comparison of calculated Raman frequencies of the Anion-Ag+ equatorial model with different levels of theory and experimental SERS data.

**SERS Ag colloid**	**Calculated B3LYP/LANL2DZ**	**Calculated B3LYP/6-311++G**+LANL2DZ(Ag)**	**Calculated CAM-B3LYP/LANL2DZ**
438	452	414	406
461	478	480	485
593	611	617	622
810	821	819	**?**
846	868	860	843
868	884	891	878
983	981	973	990
1020	1032	1017	1051
1056	1041	1034	1068
1154	1157	1153	1171
1179	1166	**?**	1182
1263	1275	1292	1286
1289	1290	**?**	1304
1355	1367	1359	1312
1373	1389	1377	1376
			1388 ?
1450	1494	1465	**?**
1470	1498	1477	1500

Table S3. Comparison of experimental and calculated frequencies for piperidine Anion linked to Ag+ and Ag_5+. The four silver atoms on the plane were kept fixed during the structural relaxation.

Table

SERS Ag colloid	Calculated Anion-Ag+ equatorial	Calculated Anion-Ag_5+ equatorial
438	452	440
461	478	469
593	611	617
810	821	815
846	868	869
868	884	881
983	981	980
1020	1032	1035
1056	1041	1048
1154	1157	1129
1179	1166	1146
1263	1275	1267
1289	1290	1290
1355	1367	1357
1373	1389	1394
1450	1488	1484
1470	1498	1490

Anion-Ag_5+ equatorial

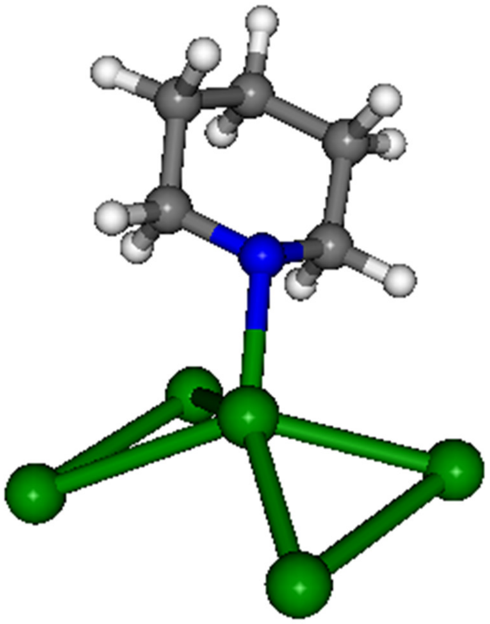

Supplement: Supplementary file 1 [file nanomaterials-12-02907-s001.zip › nanomaterials-1863481-supplementary.pdf]
